# Multi-group Analysis of Compositions of Microbiomes with Covariate Adjustments and Repeated Measures

**DOI:** 10.21203/rs.3.rs-2778207/v1

**Published:** 2023-05-02

**Authors:** Huang Lin, Shyamal Das Peddada

**Affiliations:** 1 Biostatistics and Computational Biology Branch, NIEHS, NIH, Research Triangle Park, NC, USA

## Abstract

Microbiome differential abundance analysis methods for a pair of groups are well established in the literature. However, many microbiome studies involve multiple groups, sometimes even ordered groups, such as stages of a disease, and require different types of comparisons. Standard pairwise comparisons are not only inefficient in terms of power and false discovery rates, but they may not address the scientific question of interest. In this paper, we propose a general framework for performing a wide range of multi-group analyses with covariate adjustments and repeated measures. We demonstrate the effectiveness of our methodology through two real data sets. The first example explores the effects of aridity on the soil microbiome, and the second example investigates the effects of surgical interventions on the microbiome of IBD patients.

## Introduction

Increasingly researchers are recognizing the role of the microbiome in human health and diseases^1, 2^. Accordingly, it has become a common practice for researchers to investigate differences in microbial compositions and to identify differentially abundant taxa among two or more study or experimental groups. Most existing methods for differential abundance (DA) analysis, such as ANCOM^3^, ANCOM-BC^4^, LOCOM^5^, etc., are designed for discovering differentially abundant taxa between two groups. A variety of strategies are used in the literature for performing multi-group differential abundance analysis. For example, some researchers use the above methods to analyze two groups at a time using a false discovery rate (FDR) threshold within each pairwise comparison and pool the results from all such pairwise comparisons to interpret the data. Such a strategy does not account for the fact that multiple tests and multiple pairwise comparisons are being performed when controlling for false discovery rates. Some researchers perform global beta diversity analyses for multiple groups and follow up with pairwise differential abundance analyses for groups that appear to be different. While all such approaches are intuitive and reasonable, they may not be designed to answer the specific scientific question of interest. Secondly, the statistical properties of such procedures are not well understood. For example, they may not control the overall FDR for multiple tests and multiple pairwise comparisons.

It is well recognized in gene expression studies that when there are more than two groups, the definitions of FDR depend upon the study design and the hypotheses of interest. Standard procedures, such as the BH procedure^6^, are designed for testing multiple hypotheses between two groups. When there are multiple groups, the standard concept of FDR, and methods controlling the corresponding error rates, need to be modified according to the study design and type of analyses to be performed^7–9^. A variety of multi-group studies are performed by researchers, such as: (1) *Multiple pairwise comparisons:* A dietitian may be interested in making all pairwise comparisons of the gut microbial compositions among subjects receiving diets $D_{1},D_{2}$ or $D_{3}$. Furthermore, for each pairwise comparison, $D_{1}$ vs. $D_{2},D_{1}$ vs. $D_{3}$, and $D_{2}$ vs. $D_{3}$, the goal is often to identify taxa whose abundance increased (or decreased). (2) *Multiple pairwise comparisons against a specific reference group*: Same as in scenario (1), but the investigator is only interested in a subset of pairwise comparisons. For example, compare each group with a reference group. Suppose $D_{1}$ is the standard diet, and the researcher may be interested in identifying taxa whose abundance may have increased (or decreased) for subjects receiving a new diet $D_{2}$, compared to $D_{1}$, and similarly new diet $D_{3}$, compared to $D_{1}$. (3) *Pattern analysis over ordered study groups*: In some instances, an investigator may be interested in discovering trends or patterns in abundances of taxa over ordered groups, such as the health of subjects, changes in climate, doses of a drug, and others. For instance, during normal pregnancy, women not only undergo major physiological and hormonal changes, but they also experience significant changes in their gut and vaginal microbiome^10^. These changes are necessary for maternal metabolism, immune response, and hormonal changes to support pregnancy and to provide healthy flora for babies at birth^11, 12^. In fact, the alpha diversity of the microbiome dramatically decreases temporally during pregnancy^13^. During the first trimester of a healthy pregnancy, the gut microbiota resembles that of a healthy non-pregnant woman^13^. However, as the pregnancy progresses from the first to the third trimester, there is a reduction in the relative abundance of anti-inflammatory butyrate-producing bacteria and an increase in the pro-inflammatory phylum Proteobacteria, *Bifidobacterium spp*. in the phylum Actinobacteria, and lactic acid-producing bacteria in preparation for energy demands of lactation^12^. Thus, in many scientific investigations, researchers may be interested in studying changes in the microbiome over an ordered set of conditions. The patterns of microbial abundance may not always be monotonic. They may display other shapes, such as an umbrella or an inverted umbrella with the location of the peak or trough unknown a priori. Additionally, depending upon the scientific question of interest, such as microbial changes during pregnancy, repeated measures are taken on the same subject. Although the pattern analyses mentioned here could be accomplished by conducting a sequence of pairwise tests over adjacent ordered groups, such a strategy may have lower power than a test designed for pattern analysis, as will be demonstrated in the analysis of soil aridity data described later in this paper.

The focus of this paper is to develop methodologies for performing multi-group differential abundance analyses for studies such as the above-noted ones. To the best of our knowledge, there does not exist a formal methodology for performing such analyses, with the exception of ANCOM-II^14^. While ANCOM-II considered some of the above testing problems, it does not develop a formal framework for bias correction and is heuristic. The more recent methodology LinDA^15^, which uses a model similar to the one developed in ANCOM-II, does not address the above multi-group testing problems. Thus, there is a major gap in the literature for analyzing multi-group microbiome studies, which will be filled by the methodology developed in this paper called ANCOM-BC2.

Before developing ANCOM-BC2, we first make some modifications to ANCOM-BC^4^ for testing multiple hypotheses regarding two groups which would result in a better control of FDR. This framework will then be extended to the multi-group testing problem considered in this paper. Although the ANCOM-BC methodology accounted for sample-specific bias, we now also account for taxon-specific bias. This is important because sequencing efficiencies can vary across taxa, leading to a taxon-specific bias when some taxa are preferentially measured over others during the sequencing experimental workflow. For example, gram-positive bacteria have stronger cell walls than gram-negative bacteria, making them harder to extract during the data preparation step. As a result, gram-positive bacteria may be underrepresented in the observed abundances, leading to biased results if not properly accounted for in the analysis^16^. Also, it is well-known in the analysis of other omics data that small signals are often associated with small variances. Consequently, in such cases, the value of the test statistics is inflated which potentially results in a highly significant p-value, even though the effect sizes are trivially small. Inspired by Significance Analysis of Microarrays (SAM)^17^ methodology, we regularize the variance to avoid inflated values for the test statistics and hence moderate the p-values to avoid false positives and false discoveries. Lastly, zeros are a common problem for many DA methods, including ANCOM-BC, that work with log-abundance data. Often such methods use pseudo-counts to deal with zero before taking logarithms. However, the choice of pseudo-count can impact the results of rare taxa containing excess zeros, potentially leading to an increase in false discoveries^14, 18, 19^ and inflate FDR. To mitigate this issue, we conduct a sensitivity analysis for pseudo-count addition and assign a sensitivity score to each taxon, indicating the likelihood of a false positive result for a particular taxon that is declared significant. A larger sensitivity score indicates a higher risk of a false positive, thus providing a tool for a researcher when interpreting the results.

Using constrained statistical inference-based methods^7^ and mixed directional false discovery rate (mdFDR) methods for multiple pairwise comparisons^8, 9^, along with the above-noted modifications to ANCOM-BC, we develop ANCOM-BC2 for multi-group microbiome studies in this paper. ANCOM-BC2 allows modeling covariates as well as repeated measures. Details of the method are described in the Methods Section. The performance of ANCOM-BC2 is evaluated using extensive simulation studies under a variety of settings. The results are described in the Results Section. The study designs for the simulation experiments are provided in the Supplementary Methods. ANCOM-BC2 is illustrated using two publicly available data, namely, soil microbiome data and irritable bowel disease data.

## Results

### Simulations: Continuous and Binary Exposures

In light of practical applications, we conducted simulation studies under two common scenarios, when the exposure variable is continuous or it is binary while adjusting for covariates in the model. To benchmark the performance of ANCOM-BC2, we compared it to its predecessor, ANCOM-BC^4^, which was originally developed for binary covariates, as well as two other state-of-the-art DA methods: 1) LinDA^15^, which is based on linear regression and employs centered log-ratio transformed abundance data, and 2) LOCOM^5^, a logistic regression-based approach that obviates the need for pseudo-counts and utilizes permutation methods to address overdispersion and small sample sizes.

We utilized a subset of data from the Quantitative Microbiome Project (QMP)^20^ consisting of 106 samples and 91 operational taxonomic units (OTUs) to generate simulated data. By using real data as a template, we ensured that the data-generating process did not favor our methods, enabling a fair comparison across all methods. We applied the effects of the exposure and adjusting covariates to the log abundance data based on the QMP data template. The log fold-changes (based on the natural log) of these variables ranged from −2 to 2, which corresponds to a fold-change between 0.14 to 7.4 in the original scale. To evaluate the robustness of differential abundance (DA) methods, we conducted a comprehensive simulation study that incorporated two sources of bias into the synthetic data. To account for sequencing efficiency differences, we applied a feature-specific bias that was sampled from a uniform distribution (U[0.1,1]) and applied to each taxon. Additionally, we included a sample-specific bias that ensured the presence of rare taxa (with more than 50% zeros across samples) to test the robustness of the DA methods to pseudo-count addition. The sample-specific bias was also highly correlated with the exposure of interest to assess the methods’ performance against batch effects, which are a concern in large-scale omics studies. To test the robustness of the DA methods regarding the violation of the assumption that most taxa are not differentially abundant in most DA methods, we considered four different cases of the underlying true proportion of DA taxa, ranging from 10% to 90%. For ANCOM-BC2, we used a sensitivity analysis to pseudo-count addition, setting the cutoff value at one, which means any taxon with a sensitivity score greater than one would be automatically declared not significant. To control the false discovery rate due to multiple testing, we utilized the Holm-Bonferroni method^21^ instead of the Benjamini-Hochberg (BH) procedure^6^, as it does not rely on assumptions about the dependence structure among the underlying p-values and is known to be more robust when dealing with inaccurate p-values^22^. Supplementary Figures 1 and 2 provide additional details on the simulation study design and the results obtained. More information is available in the Supplementary Methods.

**Figure 1.**
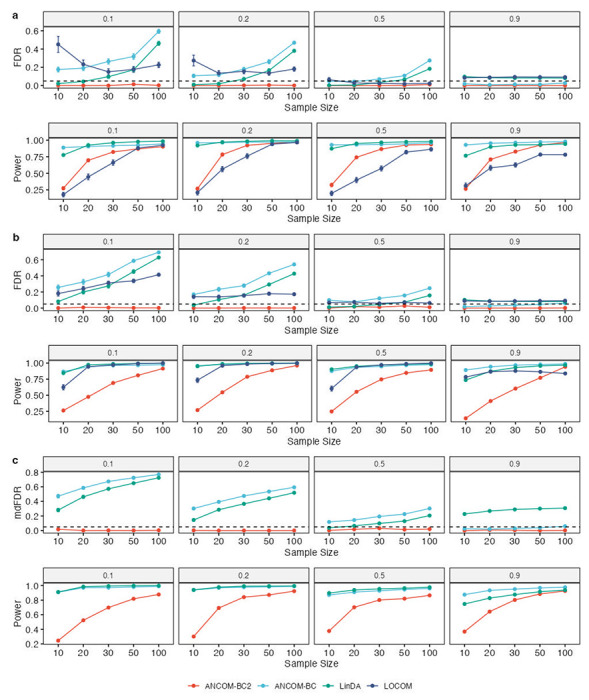
Comparisons of FDR (mdFDR) and power in identifying DA taxa in (**a**) continuous, (**b**) binary, or (**c**) categorical exposure. Synthetic data were generated based on the QMP data^20^. The X-axis shows the sample size (or sample size per group for the categorical covariate), and the Y-axis shows the FDR (mdFDR) or power. The proportion of true DA taxa is indicated in the panel title. Results are represented by the average of the corresponding measure (FDR, mdFDR, or power) ± standard errors (shown as error bars) across 100 simulation runs for each setting. The results demonstrate that ANCOM-BC2 outperformed all competing methods in terms of uniformly small FDR (mdFDR) and comparable power.

Figure 1a displays the simulation results for DA analysis with respect to a continuous exposure. ANCOM-BC2 outperformed all other methods by maintaining a FDR control under the nominal level of 0.05, even with highly dynamic microbial environments, where up to 90% of taxa were differentially abundant. Also, as expected, ANCOM-BC2 demonstrated increased power with larger sample sizes, achieving comparable power to the other methods when the sample size was relatively large (> 50). In contrast, all competing methods had substantially higher FDR than ANCOM-BC2. LOCOM had severely inflated FDR and reduced power with limited sample sizes, reaching up to 50% FDR and as low as 20% power in simulations with 10 samples and 10% of taxa being differentially abundant. ANCOM-BC and LinDA had the highest power among all methods, but both methods exhibited FDR exceeding the nominal level in most simulation scenarios. This increase in FDR correlated with the increase in sample size, suggesting a systematic bias in their test statistics due to the addition of pseudo-counts. Proper sensitivity analysis regarding pseudo-count addition is necessary to prevent random false positives and FDR inflation for rare taxa in both methods.

Simulation results for differential abundance analysis (DA) with respect to a binary exposure are shown in Figure 1b. The results are consistent with those in Figure 1a. The competing methods had substantially inflated FDR as compared to ANCOM-BC2, and their FDRs increased monotonically with the increase in sample size. ANCOM-BC2 outperformed all other methods in terms of maintaining a uniformly small FDR and reasonable power. Note that given the striking differences in FDR, it may not be reasonable to compare powers.

### Simulations: Multiple Groups

The simulation settings for multi-group comparisons were consistent with those outlined in the previous section. Similarly, we conducted a sensitivity analysis for ANCOM-BC2 with a cutoff value of one for pseudo-count addition and controlled the FDR due to multiple testing using the Holm-Bonferroni method^21^.

#### Multiple pairwise comparisons against a reference group.

In this simulation study, we evaluated the performance of ANCOM-BC2 in multiple comparisons against a reference group, where the exposure variable consisted of three groups and adjusting covariates were present. We compared ANCOM-BC2 against ANCOM-BC and LinDA. We did not include LOCOM because, currently, it is not designed for multiple groups. Figure 1c illustrates that ANCOM-BC2 effectively controlled the mdFDR^8, 9^, the combination of both Type I and the directional errors in the FDR framework (see the Methods section for details), below the nominal level of 0.05 and maintained a power greater than 0.8 when the sample size per group exceeded 30. On the other hand, neither ANCOM-BC nor LinDA controlled the FDR, highlighting the advantage of ANCOM-BC2 over existing methods in controlling mdFDR in multi-group comparisons.

#### Multiple pairwise comparisons.

**Figure 2.**
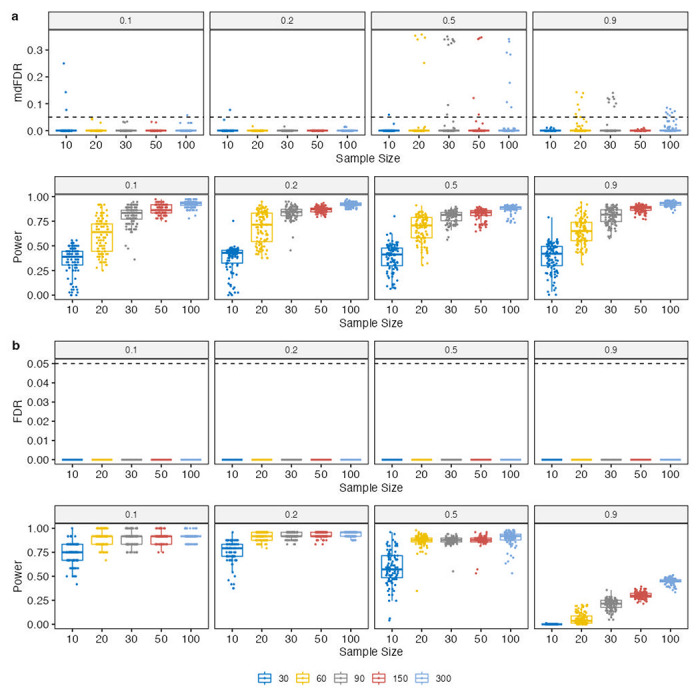
Comparisons of FDR (mdFDR) and power in identifying DA taxa in (**a**) multiple pairwise comparisons, and (**b**) pattern analysis. Synthetic data were generated based on the QMP data^20^. The X-axis shows the sample size per group, and the Y-axis shows the FDR (mdFDR) or power. The proportion of true DA taxa is indicated in the panel title. The box plot shows the distribution of the corresponding measure (FDR or power) across 100 simulation runs for each setting. Each box represents the interquartile range (IQR) of the data, with the horizontal line in the box representing the median. The whiskers extend to the furthest data point which is within 1.5 times the IQR. Any data points beyond this range are shown as individual points. Additionally, the data points are jittered to avoid overlap and provide a better visualization of the data. The results demonstrate that ANCOM-BC2 controlled FDR (mdFDR) while maintaining adequate power.

In this simulation study, we evaluated the performance of ANCOM-BC2 for all possible pairwise comparisons, rather than focusing on comparisons against a specific group. As previously mentioned, the competing methods considered in this paper are not equipped to handle pairwise comparisons in their existing forms, which led to their exclusion from this simulation study. Figure 2a demonstrates that, on average, ANCOM-BC2 effectively controlled the mdFDR well below the nominal level of 0.05 while maintaining substantial power (>0.8) when the sample size per group was not exceedingly small (n>30).

#### Pattern analysis.

Similar to pairwise comparisons, pattern analysis stands out as an important feature of ANCOM-BC2. In this simulation study, we benchmarked the scenario of a monotonically increasing pattern, where the log fold-change between the second group and the reference group, denoted as δ, ranged from 0.5 to 2, and the third group had the log fold-change equal to δ+1 with respect to the reference group. The “discovery” in the pattern analysis was defined as the identification of a taxon that was monotonically increasing across the three groups. As shown in Figure 2b, ANCOM-BC2 was successful in controlling the false discovery rate (FDR) in this simulation setting and maintained high power (>0.8) in most cases. However, under the most extreme scenario where 90% of taxa were truly differentially abundant, ANCOM-BC2 experienced a loss of power. It is important to note that in this simulation study, ANCOM-BC2 pattern analysis controlled the FDR in a very conservative manner, with an FDR equal to 0. Increasing the cutoff value for the sensitivity analysis of pseudo-count addition or using a less stringent FDR controlling method instead of the Holm-Bonferroni method could improve the power and the FDR/power tradeoff (data not shown).

### Simulations: Correlated Samples

**Figure 3.**
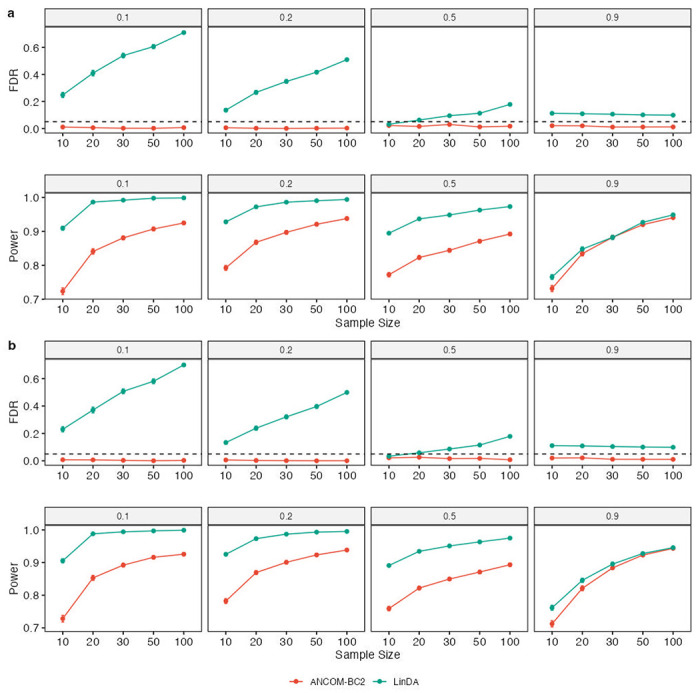
Comparisons of FDR and power in identifying DA taxa in (**a**) a random intercept model, and (**b**) a random coefficients model. Synthetic data were generated based on the QMP data^20^. The X-axis shows the sample size per group, and the Y-axis shows the FDR or power. The proportion of true DA taxa is indicated in the panel title. Results are represented by the average of the corresponding measure (FDR or power) ± standard errors (shown as error bars) across 100 simulation runs for each setting. The results demonstrate that ANCOM-BC2 outperformed LinDA in terms of uniformly small FDR and comparable power.

We also performed benchmarks comparing ANCOM-BC2 to LinDA with respect to correlated microbiome data. Note that ANCOM-BC and LOCOM were not included since neither of them is designed for correlated experimental groups, such as repeated measures. We considered two scenarios in this simulation study. The first scenario contained only a random intercept effect in the data, while the second had both random intercept and random slope effects. Both random effects had variances equal to 1, and a correlation coefficient of 0.5 was set if both were present. The exposure variable had three levels, i.e., there were three experimental groups. The simulation study included a continuous covariate. The remaining simulation settings were the same as indicated in the previous section, and we provide further details in the Supplementary Methods. The results were similar in both scenarios (Fig. 3). ANCOM-BC2 controlled the FDR well below the nominal level of 0.05 in all simulation settings while maintaining adequate power as the sample size increased. Meanwhile, although LinDA had uniformly larger power than ANCOM-BC2, it suffered inflated FDR, particularly when the sample size was large (e.g., the FDR could reach 70% when the sample size per group was 100).

### Illustration: Soil Microbiome and Aridity

The threat of climate change not only threatens the dryland ecosystems with increasing temperatures and aridity but there is a concern that some of the microbes native to such conditions may be trans-locating to other environments with the expansion of deserts and desert ecologies. There is growing literature on desert microbiome^23^. The translocation of such bacteria may affect vegetation, plant life, and human health. Most importantly, there are concerns regarding the spread of antimicrobial or antibacterial resistance-causing microbes, which may make infections harder to treat and increase the risk of spreading diseases. Accordingly, there is interest in identifying microbial species that are native to extremely hot conditions, such as hyper-arid soils.

**Figure 4.**
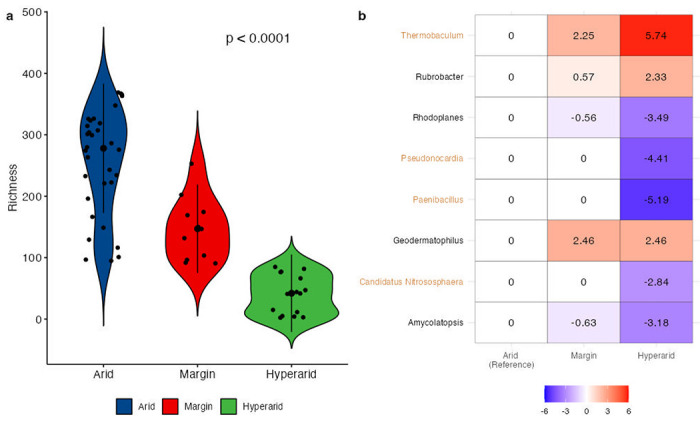
Differential abundance analysis for the impact of aridity in desert soil samples. (**a**) Violin plot of the relationship between aridity and microbial richness. The black dot in the middle of each violin represents the median value, while the black bar represents the interquartile range (IQR). The width of each violin reflects the density of data points at each value of microbial richness. Individual data points are also shown as jittered dots. The ORIOGEN software was utilized to conduct a trend test, which revealed a significant reduction in species richness with increasing aridity with a p-value <0.0001. (**b**) Heatmap of ANCOM-BC2 pattern analysis with respect to aridity. Monotonic increasing and decreasing trends were evaluated across ordered soil categories, with arid soil as the reference category. The X-axis represents soil categories, while the Y-axis displays significant genera identified by ANCOM-BC2 pattern analysis. Each cell is color-coded with blue representing reduced abundance and red representing increased abundance, with the numbers on each cell indicating the log fold-change relative to the reference group (arid group). The Holm-Bonferroni method was utilized to correct for multiple testing, and genera colored in brown on the Y-axis were found to be significant after correcting for multiple testing.

Recently, Neilson et al.^2^ investigated the differences in soil microbiomes according to soil aridity in the Atacama Desert in Chile. They classified soil into three ordered categories based on aridity, namely, arid, margin, and hyper-arid, and sequenced data from 63 sample pits from 18 sites in the desert. Since they did not conduct formal differential abundance analyses of those data, we reanalyzed those data using the ANCOM-BC2 methodology. To begin with, we conducted a trend analysis of species richness with respect to the ordered aridity categories (arid to hyper-arid) (Fig. 4a). The trend test using the ORIOGEN^24^ software yielded a p-value <0.0001, suggesting a significant loss of species richness with the increase in aridity. This finding regarding the species richness is consistent with the original publication of Neilson et al.^2^, except that we are able to provide a p-value for trend using ORIOGEN.

Next, we performed a pattern analysis to discover patterns in microbial taxa abundances over the ordered soil categories using the arid soil as the reference category. We discovered *Thermobaculum, Geodermatophilus*, and *Rubrobacter* to increase in mean abundance with the aridity of the soil (p<0.05), and among these, *Thermobaculum* had a significant trend after correcting for multiple testing with a multiple testing adjusted p-value <0.0001 (Fig. 4b). *Thermobaculum spp*. is well-known to exist in temperatures as high as 90 degrees Celsius^25^, and it is documented to contain antibacterial or antimicrobial-resistant genes^26, 27^. Similarly, the two Actinobacteria genera, *Geodermatophilus* and *Rubrobacter*, are also known to be antibacterial-resistant genera. For a review of these taxa, one may refer to Montero-Calasanz et al.^28^ and Li et al.^29^, respectively. Thus, using our proposed methodology, we discover genera that have an increase in abundance with respect to aridity and which may have antibacterial resistance properties.

Not surprisingly, nitrogen-cycling microbes are significantly diminished by increasing aridity in desert soils. For example, the presence/absence results (Supplementary Table 1) show that *Nitrobacter*, a common contributor to nitrification, along with putative broadly distributed N2 fixers *Sinorhizobium, Rhizobium*, and *Azospirillum*, were not detected in hyper-arid samples. The results of the ANCOM-BC2 pattern analysis also show that increasing aridity correlates with significant reductions in the abundance of taxa typically associated with fertile soils (Fig. 4b). Genera such as *Ca. Nitrososphaera*, which are chemolithoautotrophs and have essential biogeochemical roles as nitrifying organisms^30^; *Paenibacillus*, which contains many species that promote crop growth through nitrogen fixation, phosphate solubilization, production of the phytohormone indole-3-acetic acid (IAA), and release of siderophores that enable iron acquisition^31^; and *Pseudonocardia*, which has been reported to achieve associative nitrogen fixation and protect their hosts against soil-borne pathogenic infection^32^, monotonically decreased with aridity.

ANCOM-BC2 sensitivity analysis revealed that none of the significant taxa exceeded the threshold of 1, indicating that these discoveries are likely true positives (Supplementary Fig. 3).

### Illustration: Gut Microbial Composition of IBD Patients after Surgery

**Figure 5.**
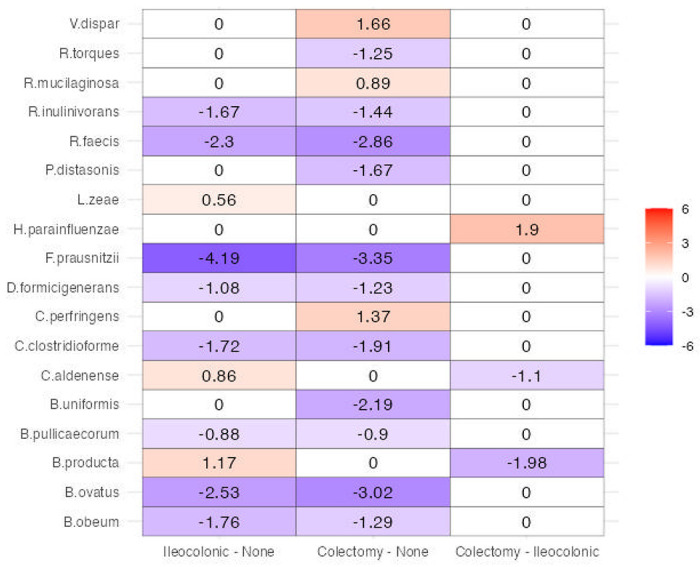
Heatmap of ANCOM-BC2 pairwise analysis for the effect of surgical resection in a cohort of patients with IBD. The analysis included multiple pairwise comparisons among the three groups: ileocolonic resection, colectomy, and no intestinal surgery, while controlling the overall mdFDR at 0.05. The X-axis represents the specific comparisons made between ileocolonic resection vs. no intestinal surgery, colectomy vs. no intestinal surgery, and ileocolonic resection vs. colectomy. The Y-axis displays the significant species identified by ANCOM-BC2. Each cell in the heatmap is color-coded with blue representing reduced abundance and red representing increased abundance, and the numbers on each cell indicate the log fold-change. The Holm-Bonferroni method was utilized to correct for multiple testing.

Numerous studies have delved into the role of the microbiome in inflammatory bowel disease (IBD); however, only a few have specifically addressed the impact of surgery on the gut microbiome and its subsequent consequences. We employ the ANCOM-BC2 to analyze a longitudinal dataset obtained from Fang et al.^33^ to investigate the changes in the gut microbiome following gastrointestinal surgery in IBD patients. The dataset utilized in this study consists of 322 stool samples collected from 125 patients. Of these, 46 patients were diagnosed with ulcerative colitis (UC) and 79 with Crohn’s disease (CD). Stool samples were obtained from each subject at approximately 6-month intervals, beginning at the baseline time point. Specifically, 21 patients provided one sample, 38 patients provided two samples, 41 patients provided three samples, 23 patients provided four samples, and 2 patients provided five samples. Of the total patient population, 87 (70.0%) had no history of intestinal surgery, while 22 CD patients had undergone ileocolonic resection, and 13 CD patients and 3 UC patients had undergone different types of colectomies. These surgeries had occurred prior to the collection of the baseline stool sample. For the purposes of this study, we focused on comparing the microbial compositions between patients who had not undergone gastrointestinal surgery, those who had undergone ileocolonic resection, and those who had undergone colectomies. We adjusted the ANCOM-BC2 model for IBD disease type (UC vs. CD) and two potential confounders, namely disease state (inactive vs. active) and antibiotic use (absent vs. present). The results are depicted in Fig. 5.

We performed multiple pairwise comparisons among the three groups controlling the overall mdFDR at 0.05 using ANCOM-BC2. Ileocoloic section is the surgical removal of the diseased section of the ileum, which is the junction area between the small and last intestines. In contrast, colectomy is the surgical removal of most or all of the large intestine. Interestingly, our analysis revealed that almost no microbial species were differentially abundant between the two surgical groups of patients, except for *H. parainfluenzae* and *C. perfringens*, which are more abundant in the colectomy group, and conversely, *C. aldense*, and *B. producta* are more abundant only in the ileocolonic section group.

We saw reduced abundances of several native gut bacterial species in patients who underwent either ileocolonic resection or colectomy compared to patients with no intestinal surgery. Specifically, These species include *Bacteroides spp*. (*ovatus* and *uniformis*), *Butyricicoccus pullicaecorum, Dorea* (*formicigenerans* and *longicatena*), *Faecalibacterium prausnitzii, Roseburia spp*. (*faecis* and *inulinivorans*), and *Ruminococcus torques*. Most of these bacterial species are involved in the production of short-chain fatty acids (SCFAs), such as butyrate, propionate, and acetate^34–40^. SCFAs play essential roles in maintaining gut health, supporting gut barrier function, possessing anti-inflammatory properties, and providing energy sources for colonocytes. Thus, although the surgical intervention was necessary for these patients, the surgery may have impacted the host immune response and health of these patients. The IBD patients who underwent any of these surgeries may require probiotics or other forms of microbial supplementation.

We also conducted an ANCOM-BC2 sensitivity analysis, revealing that none of the significant taxa exceeded a threshold of 1. This suggests that the observed findings are likely true positives (Supplementary Fig. 4).

## Discussion

In this article, we introduced a general framework called ANCOM-BC2 for performing differential abundance analysis when the exposure variable is continuous, binary, or (ordered) categorical. The proposed methodology allows for adjusting for covariates and repeated measures (longitudinal measures) while controlling for FDR or mdFDR when the exposure variable has more than two groups, and the researcher is interested in inferring if the abundance of a taxon increased or decreased for each pairwise comparison. Furthermore, using the theory of constrained statistical inference, ANCOM-BC2 allows researchers to infer patterns in microbial abundance over ordered categories of exposure variables. For example, it allows a researcher to test if a particular microbe increased (or decreased) in abundance over ordered disease categories (very healthy to least healthy). This is a unique feature of ANCOM-BC2. Lastly, ANCOM-BC2 overcomes some of the limitations of ANCOM-BC for controlling the FDR while maintaining high power, especially with the presence of rare taxa with excess zeros.

The power of ANCOM-BC2’s pattern analysis was demonstrated in the soil microbiome data analyzed in this paper. When standard pairwise analyses were performed, we only discovered *Paenibacillus* that was significantly differentially abundant across different groups (data not shown). However, using the pattern analysis, we discovered several taxa display increasing or decreasing trends over the ordered soil aridity groups. This is because, unlike pairwise comparisons, pattern analysis uses constrained inference methods, which “borrow” information from neighboring ordered groups. Thus, increasing the effective sample size and the power^7, 41, 42^.

Intrinsically, the ileocolonic section and colectomy are surgically removing different regions of the intestines, and yet based on our analysis of the IBD data, there were no significant differences in the abundance of the majority of the gut bacteria. Furthermore, the two groups of patients have similarly reduced abundances of these bacteria relative to those who did not undergo either of the two surgeries. Based on these findings, it may be reasonable to hypothesize that most species of gut microbiota are spatially uniformly distributed in the ileum and large intestines.

## Methods

### Notation

The notations described in ANCOM-BC2 methodology are summarized in Table 1.

### ANCOM-BC2 for fixed effects models

#### Model assumptions

*Assumption* 1 (Multiplicative model for observed abundances)*:*

$$O_{ij}=S_{i}C_{j}A_{ij}E_{ij}.$$

Assumption 1 indicates that, in expectation, the observed abundance of a taxon in a random sample is in constant proportion to the abundance in a unit volume of the ecosystem of the sample. This proportion can be decomposed into two parts: (1) sample-specific sampling fraction, and (2) taxon-specific sequencing efficiency.

According to Assumption 1, for non-zero observed abundance, the above multiplicative model can be transformed into an additive model by log transformation

$$o_{ij}=s_{i}+c_{j}+a_{ij}+e_{ij}.$$

*Assumption* 2 (Linear model for log abundances)*:* For each taxon $j,a_{ij},i=1,\ldots ,n$ are independently distributed, and

$$a_{ij}=b_{j}^{T}x_{i}+e_{ij}^{\left(a\right)},$$

where

1. $x_{i}={\left(1,x_{i1},x_{i2},\ldots ,x_{ip}\right)}^{T}$ are the covariates of interest (including the intercept) for the $i^{\mathrm{th}}$ sample,
2. $b_{j}={\left(b_{j0},b_{j1},b_{j2},\ldots ,b_{jp}\right)}^{T}$ are the corresponding coefficients for $x_{i}$.
3. $e_{ij}^{\left(a\right)},i=1,\ldots ,n$ are independently distributed random errors for log abundances with $E(e_{ij}^{\left(a\right)})=0,\mathrm{Var}(e_{ij}^{\left(a\right)})=\sigma_{jj}^{\left(a\right)}$.

*Assumption* 3 (Independent random error for log observed abundances)*:* Assume there are random errors, $e_{ij}^{\left(o\right)},i=1,\ldots ,n,j=1,\ldots ,d$, for log observed abundances $o_{ij}$, which are independently distributed with heteroskedasticity:

$$E\left(e_{ij}^{\left(o\right)}\right)=0,\;\mathrm{Var}\left(e_{ij}^{\left(o\right)}\right)=\sigma_{ij}^{\left(o\right)},\;e_{ij}^{\left(o\right)}⫫e_{ij}^{\left(a\right)}.$$

#### Regression framework

Based on the Assumptions 2 and 3, $o_{ij}$ can be modeled as:

(1) $$o_{ij}=s_{i}+c_{j}+{b_{j}}^{T}x_{i}+e_{ij}^{\left(a\right)}+e_{ij}^{\left(o\right)}:=s_{i}+c_{j}+{b_{j}}^{T}x_{i}+e_{ij},$$

with

$$E\left(o_{ij}\right)=s_{i}+c_{j}+{b_{j}}^{T}x_{i},\;\mathrm{Var}\left(o_{ij}\right)=\mathrm{Var}\left(e_{ij}\right)=\sigma_{jj}^{\left(a\right)}+\sigma_{ij}^{\left(o\right)}:=\sigma_{ij}^{\left(t\right)}.$$

where $\sigma_{ij}^{\left(t\right)}$ denotes the total variance.

Model (1) can also be written in a vector notation as follows:

(2) $$o_{j}=s+c_{j}1+Xb_{j}+e_{j},$$

with

$$E\left(e_{j}\right)={\left(0,\ldots ,0\right)}^{T},\;E\left(o_{j}\right)=s+c_{j}1+Xb_{j},\;\text{Cov}\left(o_{j}\right)=\left[\begin{array}{cccc} \sigma_{1j}^{\left(t\right)} & 0 & \ldots & 0\\ 0 & \sigma_{2j}^{\left(t\right)} & \ldots & 0\\ \vdots & \vdots & \ddots & \vdots \\ 0 & 0 & \ldots & \sigma_{nj}^{\left(t\right)} \end{array}\right].$$

where

1. $1=(1,1,\ldots ,1{)}^{T}$,
2. $o_{j}={\left(o_{1j},o_{2j},\ldots ,o_{nj}\right)}^{T}$,
3. $s={\left(s_{1},s_{2},\ldots ,s_{n}\right)}^{T}$,
4. $b_{j}={\left(b_{j0},b_{j1},b_{j2},\ldots ,b_{jp}\right)}^{T}$,
5. $e_{j}={\left(e_{1j},e_{2j},\ldots ,e_{nj}\right)}^{T}$,
6. $X=\left[\begin{array}{ccccc} 1 & x_{11} & x_{12} & \ldots & x_{1p}\\ 1 & x_{21} & x_{22} & \ldots & x_{2p}\\ \vdots & \vdots & \vdots & \ddots & \vdots \\ 1 & x_{n1} & x_{n2} & \ldots & x_{np} \end{array}\right]$.

It is important to note that within each sample i, for taxa $l\neq m,o_{il}$ and $o_{im}$ are not necessarily independent due to correlations between $a_{il}$ and $a_{im}$. Thus vectors $o_{l}$ and $o_{m}$ are not independent random vectors.

##### Remove the effect of taxon-specific sequencing efficiency

To eliminate the effect of $c_{j}$, we first center the log observed abundances across samples, i.e.

(3) $$y_{ij}:=o_{ij}-{\bar{o}}_{\cdot j}=\left(s_{i}-\bar{s}\right)+{b_{j}}^{T}\left(x_{i}-\bar{x}\right)+\left(e_{ij}-{\bar{e}}_{.j}\right),\;\;\;\;\;\;\;:=\theta_{i}+\beta_{j}{\;}^{T}x_{i}+\varepsilon_{ij},$$

where

1. $\beta_{jk}=b_{jk}$ for k=1,…,p, and $\beta_{j0}=\sum_{k=1}^{p}b_{jk}{\overset{‾}{x}}_{\cdot k}$,
2. $\mathrm{Var}(\varepsilon_{ij})=\frac{(n-1{)}^{2}}{n^{2}}\sigma_{ij}^{\left(t\right)}+\frac{1}{n^{2}}\sum_{i^{\prime }\neq i}\sigma_{i^{\prime }j}^{\left(t\right)}:=\sigma_{ij}$.

**Algorithm 1:** Iterative MLE

**Initialize:**
For j=1,…,d
θ←0
$y_{j}^{\left(\text{crt}\right)}\leftarrow y_{j}-\theta =y_{j}$
$\beta_{j}\leftarrow {\left(X^{T}X\right)}^{-1}X^{T}y_{j}^{\left(crt\right)}={\left(X^{T}X\right)}^{-1}X^{T}y_{j}$
**while** not converge **do**
$\theta \leftarrow \frac{1}{d}\sum_{j=1}^{d}\left(y_{j}-X\beta_{j}\right)$
$y_{j}^{\left(crt\right)}\leftarrow y_{j}-\theta$
$\beta_{j}\leftarrow {\left(X^{T}X\right)}^{-1}X^{T}y_{j}^{\left(crt\right)}$
**end while**

##### Estimation of sample-specific bias

As can be seen from (3), $\beta_{j}$ are not identifiable without determining the nuisance parameter $\theta_{i}$. We define bias-corrected log abundance $y_{ij}^{\left(crt\right)}=y_{ij}-\theta_{i}$, then the ordinary least squares (OLS) estimators of $\theta_{i}$ and $\beta_{j}$ can be obtained by iteratively solving the following equations. For ease of exposition, the algorithm is described in the vector form, i.e. $y_{j}={\left(y_{1j},y_{2j},\ldots ,y_{nj}\right)}^{T},\theta ={\left(\theta_{1},\theta_{2},\ldots ,\theta_{n}\right)}^{T}$, etc.

Upon convergence,

(4) $$\theta^{*}=\frac{1}{d}\overset{d}{\underset{j=1}{\sum }}\left(y_{j}-X\beta_{j}^{*}\right),\;y_{j}^{{\left(crt\right)}^{*}}=y_{j}-\theta^{*},\;\beta_{j}^{*}={\left(X^{T}X\right)}^{-1}X^{T}y_{j}^{{\left(crt\right)}^{*}}.$$

Therefore

(5) $$\theta^{*}=\frac{1}{d}\overset{d}{\underset{j=1}{\sum }}\left(y_{j}-X\beta_{j}^{*}\right)=\frac{1}{d}\overset{d}{\underset{j=1}{\sum }}\left(y_{j}-Py_{\text{j}}^{{\left(\text{crt}\right)}^{*}}\right)\;\;=\frac{1}{d}\overset{d}{\underset{j=1}{\sum }}\left(y_{j}-Py_{j}+P\theta^{*}\right)=\frac{1}{d}\overset{d}{\underset{j=1}{\sum }}\left[y_{j}^{\left(crt\right)}+\theta -P\left(y_{j}^{\left(crt\right)}+\theta \right)+P\theta^{*}\right]\;\;=(I-P)\theta +P\theta^{*}+\frac{1}{d}\overset{d}{\underset{j=1}{\sum }}(I-P)y_{j}^{\left(crt\right)}\;\;=(I-P)\theta +P\theta^{*}+\frac{1}{d}\overset{d}{\underset{j=1}{\sum }}\varepsilon_{j},$$

where

1. $P=X{\left(X^{T}X\right)}^{-1}X^{T}$ is the projection matrix onto 𝒞(X), the column space of X,
2. $\varepsilon_{j}=(I-P)y_{j}^{\left(crt\right)}$ with $E(\varepsilon_{j})=0$.

Rearranging (5), we see that

$$(I-P)\theta^{*}=(I-P)\theta +\frac{1}{d}\overset{d}{\underset{j=1}{\sum }}\varepsilon_{j}.$$

Taking expectations on both sides leads to

$$(I-P)\left[E\left(\theta^{*}\right)-\theta \right]=0.$$

As I−P is an orthogonal projector onto 𝒞(X), the above equation holds as long as either of the following is valid:

1. $E(\theta^{*})-\theta =0$,
2. $E(\theta^{*})-\theta \in 𝒞(X)$.

It is sufficient to consider (2) because (1) is the trivial case. If (1) were true then from (4) we deduce that there is no sample-specific effect and that $E({\beta_{j}}^{*})=\beta_{j}$. Suppose (2) is true, then there exists a vector $\delta \neq 0\in {\mathbb{R}}^{p}$, such that

(6) $$E\left(\theta^{*}\right)=\theta -X\delta .$$

Then by combining with equation (4), we have

(7) $$E\left({\beta_{j}}^{*}\right)=\delta +\beta_{j}.$$

We shall denote $\theta^{*}$ and ${\beta_{j}}^{*}$ obtained from the above iterative algorithm as preliminary estimators of θ and $\beta_{j}$, respectively. Without loss of generality, throughout this paper we assume $X^{T}X$ is a full rank matrix. If it is not a full rank matrix, then we may use any generalized inverse of $X^{T}X$ because $X{\beta_{j}}^{*}$ in (5) is invariant of the choice of generalized inverse ${\left(X^{T}X\right)}^{g}$ used in ${\beta_{j}}^{*}={\left(X^{T}X\right)}^{g}X^{T}y_{j}{\;}^{\left(\mathrm{crt}\right)}$. Thus the preliminary estimator $\theta^{*}$ provided above is invariant of the choice of generalized inverse used in deriving ${\beta_{j}}^{*}$. Furthermore, throughout this paper we are interested in testing hypothesis regarding linearly estimable parameters $A\beta_{j}$, i.e. $𝒞\left(A^{T}\right)\subset 𝒞\left(X^{T}\right)$^43^. Consequently, the estimator $A{\beta_{j}}^{*}$ is invariant of the generalized inverse used in the estimation of ${\beta_{j}}^{*}$. Hence throughout this text, for simplicity of exposition, we shall assume $X^{T}X$ is of full rank.

For each taxon j=1,…,d, by (7), ${\beta_{j}}^{*}$ is a biased estimator if δ≠0. Suppose we wish test the following hypothesis

$$H_{0}:A\beta_{j}=A{\beta_{j}}^{0},\;H_{1}:A\beta_{j}\neq A{\beta_{j}}^{0}.$$

Under the null hypothesis, $E(A{\beta_{j}}^{*})-A{\beta_{j}}^{0}=A\delta \neq 0$ and hence biased. The next step is to estimate this bias δ and accordingly modify the estimator $A{\beta_{j}}^{*}$ so that the resulting estimator is asymptotically centered at $A{\beta_{j}}^{0}$ under the null hypothesis and hence the test statistic is asymptotically centered at zero.

First we make the following observations. Since $E({\beta_{j}}^{*})=\delta +\beta_{j}$, we note that as n→∞, for finite dimension d,

(8) $${\sum_{j}}^{-\frac{1}{2}}\left({\beta_{j}}^{*}-\left(\delta +\beta_{j}\right)\right)\to_{d}N_{p}(0,I),$$

where

(9) $$\sum_{j}=\underset{n\to \infty }{\text{lim}}{\left(X^{T}X\right)}^{-1}\left(\overset{n}{\underset{i=1}{\sum }}\sigma_{ij}^{2}x_{i}{x_{i}}^{T}\right){\left(X^{T}X\right)}^{-1}.$$

Since

$$E\left(\theta^{*}+X{\beta_{j}}^{*}\right)=\theta -X\delta +X\left(\delta +\beta_{j}\right)=\theta +X\beta_{j},$$

i.e. $\theta^{*}+X{\beta_{j}}^{*}$ is an unbiased estimator of $\theta +X\beta_{j}$, hence a possible estimator of ${\text{Σ}}_{j}$ is given by

(10) $${\hat{\text{Σ}}}_{j}={\left(X^{T}X\right)}^{-1}\left(\overset{n}{\underset{i=1}{\sum }}{\left(y_{ij}-\theta_{i}^{*}-{\beta_{j}}^{*T}x_{i}\right)}^{2}x_{i}{x_{i}}^{T}\right){\left(X^{T}X\right)}^{-1}.$$

Under some mild regularity conditions^44^, with finite d, we have the following consistency result

(11) $$n\left({\hat{\text{Σ}}}_{j}-{\text{Σ}}_{j}\right)\to_{p}0,\;\text{as}\;n\to \infty .$$

Therefore, replacing $\Sigma_{j}$ with ${\overset{ˆ}{\Sigma }}_{j}$ in (8) and appealing to Slutsky’s theorem, we have

$${\hat{\text{Σ}}}_{j}^{-\frac{1}{2}}\left(\beta_{j}^{*}-\left(\delta +\beta_{j}\right)\right)\to_{d}N_{p}(0,I),\;\text{as}\;n\to \infty .$$

By (9) and (11), under some mild regularity conditions^44^, for finite d, we obtain

$${\hat{\text{Σ}}}_{j}\to_{p}0,\;\text{as}\;n\to \infty .$$

Consequently,

(12) $${\beta_{j}}^{*}\to_{p}\delta +\beta_{j},\;\text{as}\;n\to \infty .$$

The above observation regarding the convergence of ${\beta_{j}}^{*}$ plays a critical role in the following. Since the sampling fraction is constant for all taxa within a sample, we pool information across taxa within each sample when estimating δ. We model each taxon abundance using the following Gaussian mixture model. For the $j^{\text{th}}$ taxon and the $k^{\text{th}}$ covariate, let $C_{0}$ denote the set of taxa that are not differentially abundant with respect to $x_{ik}$, i.e. $C_{0}=\{j\in (1,2,\ldots ,d):\beta_{jk}=0\},C_{1}$ denote the set of taxa whose abundance decreases with $x_{ik}$, i.e. $C_{1}=\{j\in (1,2,\ldots ,d):\beta_{jk}<0\}$, and let $C_{2}$ denote the set of taxa whose abundance increases with $x_{ik}$, i.e. $C_{2}=\{j\in (1,2,\ldots ,d):\beta_{jk}>0\}$. Let $\pi_{r}$ denote the probability that a taxon belongs to set $C_{r},r=0,1,2$. For simplicity of estimation of parameters, similar to GEE, we shall assume that $\beta_{jk}^{*},j=1,2,\ldots ,d$, are independently distributed. As commonly done in the analyses of various ’omics data, we ignore the underlying correlation structure when estimating δ. Thus, we model the distribution of $\beta_{jk}^{*}$ by Gaussian mixture model as follows:

(13) $$f\left(\beta_{jk}^{*}\right)=\pi_{0}\phi \left(\frac{\beta_{jk}^{*}-\delta_{k}}{v_{j0}}\right)+\pi_{1}\phi \left(\frac{\beta_{jk}^{*}-\left(\delta_{k}+l_{1}\right)}{v_{j1}}\right)+\pi_{2}\phi \left(\frac{\beta_{jk}^{*}-\left(\delta_{k}+l_{2}\right)}{v_{j2}}\right),$$

where

1. ϕ is the standard normal density function,
2. $\delta_{k},\delta_{k}+l_{1}$, and $\delta_{k}+l_{2}$ are means for $\beta_{jk}^{*}|C_{0},\beta_{jk}^{*}|C_{1}$, and $\beta_{jk}^{*}|C_{2}$, respectively. $l_{1}<0,l_{2}>0$,
3. $v_{j0},v_{j1}$, and $v_{j2}$ are variances of $\beta_{jk}^{*}|C_{0},\beta_{jk}^{*}|C_{1}$, and $\beta_{jk}^{*}|C_{2}$, respectively.

Note that instead of fitting a multivariate Gaussian mixture model for all covariates together, we choose to fit a univariate Gaussian mixture model repeatedly for every single covariate. This repetition is simply because the sets of taxa $\left\{C_{0},C_{1},C_{2}\right\}$ are not necessarily the same for different covariates. Also, note that for a categorical covariate of s levels, it contains s coefficients, e.g. $\beta_{j1},\ldots ,\beta_{js}$, and we shall fit the Gaussian mixture model for these s coefficients separately.

For computational simplicity, we assume that $v_{j1}>v_{j0},v_{j2}>v_{j0}$. Thus, Without loss of generality for $\kappa_{1},\kappa_{2}>0$, let $v_{j1}=v_{j0}+\kappa_{1}$ and $v_{j2}=v_{j0}+\kappa_{2}$. While this assumption is not a requirement for our method, it is reasonable to assume that variability among differentially abundant taxa is larger than that among the null taxa. By making this assumption, we simplify the computation.

Assuming samples are independent, we begin by first estimating $v_{j0}^{2}=\mathrm{Var}(\beta_{jk}^{*})$. Note that $v_{j0}^{2}$ is the function of heteroscedastic variances, a consistent estimator of $v_{j0}^{2}$, which we refer to as ${\overset{ˆ}{v}}_{j0}^{2}$, is the $k^{\text{th}}$ diagonal element of ${\overset{ˆ}{\Sigma }}_{j}$ stated in (10). In all future calculations, we plug in ${\overset{ˆ}{v}}_{j0}^{2}$ for $v_{j0}^{2}$. This is similar in spirit to many statistical procedures involving nuisance parameters. The following lemma is useful in the sequel.

*Lemma* 1 (Introducing the latent variable in calculating log-likelihood^45^)*:*

$$\text{log}\;f(x|\theta )=E_{f(z|x,\theta )}[\text{log}\;f(z|\theta )+\text{log}\;f(x|z,\theta )].$$

Let $\Theta ={\left(\delta_{k},\pi_{1},\pi_{2},\pi_{3},l_{1},l_{2},\kappa_{1},\kappa_{2}\right)}^{T}$ denote the set of unknown parameters, then for each taxon the log-likelihood can be reformulated using Lemma 1, as follows:

(14) $$\text{Θ}\leftarrow \underset{\text{Θ}}{\text{arg max}}\overset{d}{\underset{j=1}{\sum }}\overset{2}{\underset{r=0}{\sum }}p_{r,j}\left[\text{log}\;\mathrm{Pr}\left(j\in C_{r}\right)+\text{log}\;f\left(\beta_{jk}|j\in C_{r}\right)\right].$$

Then the E-M algorithm is described as follows:

- E-step: Compute conditional probabilities of latent variables. Define $p_{r,j}=\mathrm{Pr}(j\in C_{r}|\beta_{jk},\Theta )=\frac{\pi_{r}\phi (\frac{\beta_{jk}-\left(\delta_{k}+l_{r}\right)}{v_{j}})}{\sum_{r}\pi_{r}\phi (\frac{\beta_{jk}-\left(\delta_{k}+l_{r}\right)}{v_{jr}})},r=0,1,2;j=1,\ldots ,d$, which are conditional probabilities representing the probability that an observed value follows each distribution. Note that $l_{0}=0$.
- M-step: Maximize the likelihood function with respect to the parameters, given the conditional probabilities.

We shall denote the resulting estimator of $\delta_{k}$ upon convergence of the algorithm by ${\overset{ˆ}{\delta }}_{k}^{EM}$.

As stated in Lin and Peddada^4^, compared to ${\overset{ˆ}{v}}_{j0}^{2}$, the variance and covariance contributed by ${\overset{ˆ}{\delta }}_{k}^{EM}$ is negligible when the number of non-differentially abundant taxa is large, such as when analyzing the microbiome data at the OTU/ASV or species level of the phylogenetic tree.

The above procedure is applied to every $\beta_{jk},k=1,\ldots ,p$, eventually, we obtain the estimator of δ as

(15) $${\hat{\delta }}^{EM}={\left({\hat{\delta }}_{1}^{EM},{\hat{\delta }}_{2}^{EM},\ldots ,{\hat{\delta }}_{p}^{EM}\right)}^{T}.$$

Therefore, the final estimator of $\beta_{j}$ is defined as

(16) $${\hat{\beta }}_{j}={\beta_{j}}^{*}-{\hat{\delta }}^{EM},$$

with

(17) $${\hat{\beta }}_{j}\to_{p}\beta_{j},\;\text{as}\;n\to \infty ,$$

given that ${\overset{ˆ}{\delta }}^{EM}$ is a good approximation of δ.

The estimation procedure is summarized in Algorithm 2.

**Algorithm 2:** E-M algorithm

1:	**Input:**
	${\beta_{j}}^{*},\sum_{j},j=1,\ldots ,d$
2:	**procedure** $E-M\left({\beta_{j}}^{*},\sum_{j}\right)$
3:	**return** ${\hat{\delta }}_{k}^{EM},k=1,\ldots ,p$
4:	**end procedure**
5:	**for** k=1,…,p **do**
6:	${\hat{\beta }}_{jk}\leftarrow \beta_{jk}^{*}-{\hat{\delta }}_{k}^{EM}$
7:	**end for**

For taxon j, we now describe our methodology for testing the following hypotheses

$$H_{0}:A\beta_{j}=A{\beta_{j}}^{0},\;H_{1}:A\beta_{j}\neq A{\beta_{j}}^{0}.$$

From Slutsky’s theorem, as n→∞, the following test statistic is approximately central chi-square distributed under the null hypothesis

$$W_{j}={\left(A{\hat{\beta }}_{j}-A{\beta_{j}}^{0}\right)}^{T}{\left(A{\hat{\text{Σ}}}_{j}A^{T}\right)}^{-1}\left(A{\hat{\beta }}_{j}-A{\beta_{j}}^{0}\right)\;\;={\left(A{\beta_{j}}^{*}-A{\hat{\delta }}^{EM}-A{\beta_{j}}^{0}\right)}^{T}{\left(A{\hat{\text{Σ}}}_{j}A^{T}\right)}^{-1}\left(A{\beta_{j}}^{*}-A{\hat{\delta }}^{EM}-A{\beta_{j}}^{0}\right)\;\;\to_{d}\chi_{q}^{2},$$

where q=rank(A).

To control the FDR due to multiple testing, we recommend applying Holm-Bonferroni method^21^ instead of Benjamini-Hochberg (BH) procedure^6^ because the Holm-Bonferroni method does not require any assumptions regarding the dependence structure in the underlying p-values, and is also known to be a better method to control FDR when p-values are not accurate^22^.

##### Sampling-specific biases estimation

After obtaining ${\overset{ˆ}{\delta }}^{EM}$, the estimator of sample-specific biases θ is defined as follows:

(18) $$\hat{\theta }=\frac{1}{d}\overset{d}{\underset{j=1}{\sum }}\left(y_{j}-X{\hat{\beta }}_{j}\right).$$

Let $\Sigma^{\left(i\right)}={\left[\sigma_{lm}^{\left(i\right)}\right]}_{l,m=1,\ldots ,d}$ denote the d×d covariance matrix of $\varepsilon^{\left(i\right)}={\left(\varepsilon_{1}^{\left(i\right)},\varepsilon_{2}^{\left(i\right)},\ldots ,\varepsilon_{d}^{\left(i\right)}\right)}^{T}$, where $\sigma_{lm}^{\left(i\right)}$ is the $(l,m{)}^{th}$ element of $\Sigma^{\left(i\right)}$ and $\sigma_{jj}^{\left(i\right)}$ is the $j^{th}$ diagonal element of $\Sigma^{\left(i\right)}$. Furthermore, suppose

*Assumption* 4 (Sparse correlations among taxa)*:*

$$\sigma_{jj}^{\left(i\right)}<K<\infty ,\;\frac{\sum_{l\neq m}^{d}\sigma_{lm}^{\left(i\right)}}{d^{2}}=o(1).$$

From Assumption 4, we have

$$0\leq 1^{T}{\text{Σ}}^{\left(i\right)}1=\overset{d}{\underset{l=1}{\sum }}\overset{d}{\underset{m=1}{\sum }}\sigma_{lm}^{\left(i\right)}=\overset{d}{\underset{j=1}{\sum }}\sigma_{jj}^{\left(i\right)}+\overset{d}{\underset{l\neq m}{\sum }}\sigma_{lm}^{\left(i\right)}\leq dK+\overset{d}{\underset{l\neq m}{\sum }}\sigma_{lm}^{\left(i\right)}.$$

Hence

$$0\leq \frac{1^{T}{\text{Σ}}^{\left(i\right)}1}{d^{2}}\leq \frac{K}{d}+\frac{\sum_{l\neq m}^{d}\sigma_{lm}^{\left(i\right)}}{d^{2}}=o(1).$$

Thus, for each taxon j=1,2,…,d, we have

(19) $$\frac{1}{d}\overset{d}{\underset{j=1}{\sum }}\left(y_{j}-\left(\theta +X\beta_{j}\right)\right)\to_{p}0,\;\text{as}\;d\to \infty .$$

Therefore, according to (17) and (19), as both n,d→∞,

(20) $$\hat{\theta }\to \theta .$$

*Remark* 1. Regularization of variance: To avoid the spurious detection of significance due to extremely small standard errors, particularly for rare taxa, we incorporated a small positive constant in the denominator of the ANCOM-BC2 test statistic for each taxon. This approach was inspired by the Significance Analysis of Microarrays (SAM) methodology^17^. Specifically, the regularization factor was set as the 5th percentile of the distribution of standard errors for each fixed effect, unless otherwise specified.

*Remark* 2. Sensitivity analysis for the pseudo-count addition: To mitigate the risk of inflated false positive rates resulting from the choice of pseudo-count in ANCOM-BC2, we conducted a sensitivity analysis to assess the impact of varying pseudo-count values on differential abundance results. This is particularly important, as several studies have shown that the choice of pseudo-count can significantly influence the results of differential abundance analysis methods^18, 19^. Specifically, we surveyed a range of pseudo-count values from 0 to 1 with 0.01 increments, and a sensitivity score was assigned to each taxon to evaluate its sensitivity to the choice of pseudo-count. The sensitivity score was defined as the standard deviation of the resulting negative log p-values, with a larger score indicating greater sensitivity to the pseudo-count choice. Consequently, a significant result for a taxon with a high sensitivity score is likely to be a false positive.

#### Multi-group comparison

In some applications, for a given taxon, researchers are interested in drawing inferences regarding differential abundance among different pairs of experimental groups. We refer to this kind of problem as a multi-group comparison problem, and extra caution needs to be exercised to correct p-values due to multiple comparisons. For simplicity, we drop the subscript j (taxon index) in the following discussions.

##### Global test

For a given taxon and a total of g+1 experimental groups (including the reference group), researchers may want to test whether there exists at least one group that is significantly different from others. For ease of exposition, we split the covariates X into two parts, where $X_{1}$ stands for the group assignment, and $X_{2}$ denotes the remaining covariates. Note that the difference of group effects against the reference group is estimable, while the individual group effect is not. For simplicity, in the discussions of multi-group comparisons among group 0 to group g, we assume group 0 is the reference group. We use $\beta_{k},k=1,\ldots ,g$ to denote the group effect, but notice that it actually estimates $\beta_{k}-\beta_{0}$. We rewrite the model stated in (3) as

(21) $$y=\theta +X_{1}\beta +X_{2}\gamma +\varepsilon ,$$

where

1. θ is the sample-specific bias,
2. β is the vector of group effects (as compared to group 0) of the order g×1,
3. $X_{1}$ is the design matrix of the order n×g consisting of 0s and 1s,
4. $X_{2}$ is the known matrix of other covariates (including the intercept) of the order n×(p−g+1) with the corresponding regression parameter vector γ of the order (p−g+1)×1.

The global test intends to test

$$H_{0}:\cap_{k\in \{1,\ldots ,g\}}\beta_{k}=0,\;H_{1}:\cup_{k\in \{1,\ldots ,g\}}\beta_{k}\neq 0,$$

which can be reformulated as

$$H_{0}:A\beta =0,\;H_{1}:A\beta \neq 0,$$

where

$$A=I_{g}=\left[\begin{array}{ccccc} 1 & 0 & 0 & \ldots & 0\\ 0 & 1 & 0 & \ldots & 0\\ \vdots & \vdots & \ddots & \vdots & \vdots \\ 0 & 0 & \ldots & 0 & 1 \end{array}\right]$$

with the test statistic

$$W={\left(A\hat{\beta }\right)}^{T}{\left(A{\hat{\text{Σ}}}^{\left(g\right)}A^{T}\right)}^{-1}(A\hat{\beta })\to_{d}\chi_{g}^{2},\;\text{as}\;n\to \infty ,$$

where ${\overset{ˆ}{\Sigma }}^{\left(g\right)}$ is the corresponding sub-matrix of $\overset{ˆ}{\Sigma }$ defined in equation (10).

Similarly, to control the FDR due to multiple testing, we recommend applying Holm-Bonferroni method^21^ instead of Benjamini-Hochberg (BH) procedure^6^ due to the underlying complex dependence structure between taxa.

*Example* 0.1. Suppose there are 3 groups, namely, groups 0 (reference), 1, and 2, and no other covariates. For each sample i,i=1,…,n, we have:

$$y_{i}=\theta_{i}+\mu +\beta_{1}I\{\text{group}=1\}+\beta_{2}I\{\text{group}=2\}+\varepsilon_{i}.$$

To test whether there is at least one group among 0, 1, and 2, that is significantly different from others, we would like to test:

$$H_{0}:\beta_{1}=\beta_{2}=0,\;H_{1}:\beta_{1}\neq 0\cup \beta_{2}\neq 0,$$

which is the same as testing:

$$H_{0}:A\beta =0,\;H_{1}:A\beta \neq 0,$$

where $A=\left[\begin{array}{ll} 1 & 0\\ 0 & 1 \end{array}\right]$, and $\beta ={\left(\beta_{1},\beta_{2}\right)}^{T}$.

##### Multiple pairwise comparisons

If we are interested in knowing whether the abundance increased or decreased between various pairs of groups, then it amounts to testing the following hypotheses:

$$H_{0,k,k^{\prime }}:\beta_{k}=\beta_{k^{\prime }}\;H_{1,k,k^{\prime }}:\left\{\beta_{k}<\beta_{k^{\prime }}\right\}\cup^{\text{​}}\left\{\beta_{k}>\beta_{k^{\prime }}\right\},$$

where $k\neq k^{\prime }\in \{1,\ldots ,g\}$. Denote the test statistic for a given pairwise comparison as

$$W_{kk^{\prime }}=\frac{{\hat{\beta }}_{k}-{\hat{\beta }}_{k^{\prime }}}{\sqrt{\hat{\text{Var}}\left({\hat{\beta }}_{k}\right)+\hat{\text{Var}}\left({\hat{\beta }}_{k^{\prime }}\right)}}\to_{d}N(0,1),\;\text{as}\;n\to \infty ,$$

where $\hat{\mathrm{Var}}({\overset{ˆ}{\beta }}_{k}),\hat{\mathrm{Var}}({\overset{ˆ}{\beta }}_{k^{\prime }})$ are the $k^{\text{th}}$ and $k^{\prime \mathrm{th}}$ diagonal elements of ${\overset{ˆ}{\Sigma }}^{\left(g\right)}$, respectively. Thus, the raw p-value for comparing group k and group $k^{\prime }$ is defined as:

$$p_{kk^{\prime }}=2\left[1-\phi \left(\left|W_{kk^{\prime }}\right|\right)\right].$$

For comparing with the reference group (group 0), the hypotheses become:

$$H_{0,k}:\beta_{k}=0\;H_{1,k}:\left\{\beta_{k}<0\right\}\cup \left\{\beta_{k}>0\right\}.$$

We also replace ${\overset{ˆ}{\beta }}_{k^{\prime }}$ and $\hat{\mathrm{Var}}({\overset{ˆ}{\beta }}_{k^{\prime }})$ with 0s in the test statistic.

Note that the null and alternative hypotheses for the global test are denoted as $H_{0}$ and $H_{1}$, a Type I error might occur due to wrongly rejecting $H_{0}$ or correctly rejecting $H_{0}$ but wrongly rejecting $H_{0,k,k^{\prime }}$. A directional error might occur due to correctly rejecting $H_{0}$ but wrong assignment of the direction between $\beta_{k}$ and $\beta_{k^{\prime }}$ while correctly rejecting $H_{0,k,k^{\prime }}$. In this case, we need to control the error rate combining both Type I and the directional errors in the FDR framework, which is referred to as mixed directional FDR (mdFDR)^8, 9^.

*Definition* 1 (mdFDR): Let V(j) denote the indicator function of at least one Type I error or directional error committed, i.e.


$$V(j)=\{\begin{array}{ll} 1 & \text{if Type I or directional error occurs}\\ 0 & \text{otherwise}. \end{array}$$

Then, mdFDR is defined as the expected proportion of Type I and directional errors among all discovered taxa.

$$mdFDR=E\left(\frac{\sum_{j=1}^{d}V(j)}{\text{max}(R,1)}\right),$$

where R denote the number of taxa discovered.

To control the mdFDR for all pairwise tests, we adopt the general mdFDR controlling procedure^9^, and do the following:

1. Apply global test method stated above to obtain the p-value for each taxon. We denote these p-values as screening p-values. Apply BH procedure to identify taxa that are differentially abundant in at least one pairwise comparison. Let R denote the number of taxa discovered.
2. For each taxon discovered in step 1, apply any mixed directional family wise error (mdFWER) controlling procedure, such as Holm-Bonferroni (default), Hochberg, etc., to the pairwise p-values $\left(p_{kk^{\prime }}\right)$ at level Rα/d.
3. For a given taxon discovered in step 1, if a pairwise hypothesis is rejected in step 2, then we declare $\beta_{k}<\beta_{k^{\prime }}$ or $\beta_{k}>\beta_{k^{\prime }}$ according to $W_{kk^{\prime }}<0\;\mathrm{or}\;>0$.

It has been proved that under the assumption of independence of p-values obtained from the global test, the mdFDR of the above procedure is strongly controlled at level α^9^.

*Example* 0.2. Suppose there are 3 groups, namely, groups 0 (reference), 1, and 2, and no other covariates. For each sample i,i=1,…,n, we have:

$$y_{i}=\theta_{i}+\mu +\beta_{1}I\{\mathrm{group}=1\}+\beta_{2}I\{\mathrm{group}=2\}+\varepsilon_{i}.$$

To test whether the taxon is differentially abundant between group 1 and 0 (reference), we would like to test:

$$H_{0}:\beta_{1}=0,\;H_{1}:\left\{\beta_{1}<0\right\}\cup \left\{\beta_{1}>0\right\},$$

with the test statistic:

$$W_{10}=\frac{{\hat{\beta }}_{1}}{\sqrt{\hat{\text{Var}}\left({\hat{\beta }}_{1}\right)}}.$$

Additionally, if we want to test whether the taxon is differentially abundant between group 1 and 2:

$$H_{0}:\beta_{1}=\beta_{2},\;H_{1}:\left\{\beta_{1}<\beta_{2}\right\}\cup \left\{\beta_{1}>\beta_{2}\right\}.$$

The test statistic is:

$$W_{12}=\frac{{\hat{\beta }}_{1}-{\hat{\beta }}_{2}}{\sqrt{\hat{\text{Var}}\left({\hat{\beta }}_{1}\right)+\hat{\text{Var}}\left({\hat{\beta }}_{2}\right)}}.$$

##### Test against a specific group

Often researchers are interested in knowing whether the abundance increased or decreased in an ecosystem relative a pre-specified group, say the control group. Again, assume group 0 is the reference group and $\beta_{0}=0$, then one may be interested in testing the following hypotheses:

$$H_{0,k}:\beta_{k}=0,\;H_{1,k}:\left\{\beta_{k}<0\right\}\cup^{\text{​}}\left\{\beta_{k}>0\right\},$$

where k∈{1,…,g}.

As before, the pairwise test statistic is defined as follows:

$$W_{k}=\frac{{\hat{\beta }}_{k}}{\sqrt{\hat{\text{Var}}\left({\hat{\beta }}_{k}\right)}}\to_{d}N(0,1),\;\text{as}\;n\to \infty ,$$

where $\hat{\mathrm{Var}}({\overset{ˆ}{\beta }}_{k})$ is the $k^{th}$ diagonal elements of ${\overset{ˆ}{\Sigma }}^{\left(g\right)}$. Thus, the raw p-value for comparing group k and group 1 is defined as

$$p_{k}=2\left[1-\phi \left(\left|W_{k}\right|\right)\right].$$

Likewise, we apply the mdFDR controlling procedure for all pairwise tests. To improve power, we modify the global test mentioned earlier to a Dunnet-based^46–48^ test as described below:

1. The test statistic $W=\underset{k\in \{1,\ldots ,g\}}{\max}\left|W_{k}\right|$,
2. Generate $W_{k}^{\left(b\right)}∼N(0,1),k=1,\ldots ,g$.
3. Compute $W^{\left(b\right)}=\underset{k\in \{1,\ldots ,g\}}{\max}|W_{k}^{\left(b\right)}|$.
4. Repeat the above steps B times, we get the null distribution of W.

The screening p-value is calculated as:

$$p=\frac{1}{B}\overset{B}{\underset{b=1}{\sum }}I\left(W^{\left(b\right)}>W\right).$$

#### Pattern analysis

When the experimental groups are ordered naturally, such as doses of exposure or duration of exposure or stages of a disease, etc., for a given taxon, researchers may be interested in testing whether the abundance of the taxon is changing with the ordered experimental groups according to some specific pattern. Thus, the null and alternative hypotheses one wants to test become (assume group 0 is the reference group):

$$H_{0}:\beta_{1}=\beta_{2}=\ldots =\beta_{g}=0,\;H_{1}:\beta ={\left(\beta_{1},\ldots ,\beta_{g}\right)}^{T}\in \mathbb{C},$$

where ℂ is one or a collection of patterns. Examples of patterns are given below.

*Example* 0.3 (Simple order).


(22) $${\mathbb{C}}_{1}=\left\{0\leq \beta_{1}\leq \beta_{2}\leq \ldots \leq \beta_{g}\right\}\;\text{with at least one strict inequality.}$$

*Example* 0.4 (Tree order).


(23) $${\mathbb{C}}_{2}=\left\{\beta_{k}\geq 0,k=1,\ldots ,g\right\}\;\text{with at least one strict inequality.}$$

*Example* 0.5 (Umbrella order).


(24) $${\mathbb{C}}_{4}=\left\{0\leq \beta_{1}\leq \ldots \leq \beta_{k-1}\leq \beta_{k}\geq \beta_{k+1}\ldots \geq \beta_{g}\right\}\;\text{with at least one strict inequality.}$$

Estimation of β under a certain pattern (constraint) can be obtained by solving the following convex optimization problem^49^:

(25) $${\hat{\beta }}^{\text{opt}}=\underset{\beta \in \mathbb{C}}{\text{arg min}}{\left(\hat{\beta }-\beta \right)}^{T}{\hat{\text{Σ}}}^{{\left(g\right)}^{-1}}\left(\hat{\beta }-\beta \right),$$

where ${\overset{ˆ}{\Sigma }}^{\left(g\right)}$ is the corresponding sub-matrix of $\overset{ˆ}{\Sigma }$ defined in (10). The solution to (25) can be numerically obtained by using a suitable convex optimization algorithm, such as CVRX^50^.

*Example* 0.6. Suppose there are 3 groups, namely, groups 0 (reference), 1, and 2, and no other covariates. For each sample i,i=1,…,n, we have:

$$y_{i}=\theta_{i}+\mu +\beta_{1}I\{\text{group}=1\}+\beta_{2}I\{\text{group}=2\}+\varepsilon_{i}.$$

To test whether the group effect is monotonically increasing, we would like to test:

$$H_{0}:\beta_{1}=\beta_{2}=0,\;H_{1}:\beta \in \mathbb{C}=\left\{0\leq \beta_{1}\leq \beta_{2}\right\},\;\text{with at least one strict inequality.}$$

The estimation of β under ℂ can be obtained by solving:

$${\hat{\beta }}^{\text{opt}}=\underset{\beta \in {\mathbb{R}}^{2}}{\text{arg min}}{\left(\hat{\beta }-\beta \right)}^{T}{\hat{\text{Σ}}}^{{\left(g\right)}^{-1}}\left(\hat{\beta }-\beta \right),\;\text{s.t.}\;A\beta \geq 0,$$

where $A=\left[\begin{array}{cc} 1 & 0\\ -1 & 1 \end{array}\right]$, and $\beta ={\left(\beta_{1},\beta_{2}\right)}^{T}$.

Once the constrained estimator is obtained, there exist a variety of options to test the above hypotheses. For example, one may consider William’s type of statistic^51^. We adopt the following definitions from Peddada et al. (2002)^7^ to facilitate the construction of the test statistic.

*Definition* 2 (Linked parameters)*:* Two parameters in a given pattern are said to be linked if the inequality between them is specified *a priori*.

*Definition* 3 (Nodal parameter)*:* For a given pattern, a parameter is said to be nodal if it is linked with every other parameter in the profile.

For example, every parameter is a nodal parameter in ${\mathbb{C}}_{1}$; no nodal parameter in ${\mathbb{C}}_{2}$; and $\beta_{k}$ is the only nodal parameter in ${\mathbb{C}}_{3}$.

*Definition* 4 (Norm of maximum difference)*:* Define the norm $l_{\infty }(\mathbb{C})$ of pattern ℂ as the maximum difference between the estimates of two linked parameters.

For example, $l_{\infty }({\mathbb{C}}_{3})=\max\{{\overset{ˆ}{\beta }}_{k},{\overset{ˆ}{\beta }}_{k}-{\overset{ˆ}{\beta }}_{g}\}$.

Given a collection of potential patterns, ${\mathbb{C}}_{1},{\mathbb{C}}_{2},\ldots ,{\mathbb{C}}_{T}$, the William’s type of test statistic is defined as:

$$W=\text{max}\left\{l_{\infty }\left({\mathbb{C}}_{t}\right),t=1,\ldots ,T\right\},\;\text{with}\;t^{opt}=\text{arg max}\left\{l_{\infty }\left({\mathbb{C}}_{t}\right),t=1,\ldots ,T\right\},$$

where $t^{\text{opt}}$ is regarded as the optimal pattern for the microbial abundance of a specific taxon.

Under null hypothesis, the expectations for ${\overset{ˆ}{\beta }}_{k},k=1,\ldots ,g$ are 0s; thus, we can construct the null distribution of W as follows:

1. Generate ${\overset{ˆ}{\beta }}_{k}^{\left(b\right)}∼\sqrt{\hat{\mathrm{Var}}({\overset{ˆ}{\beta }}_{k})}N(0,1),l=1,\ldots ,g$.
2. Obtain constrained regression estimators for ${\overset{ˆ}{\beta }}_{k}^{opt,(b)}$ using the convex optimization problem described above.
3. Compute $W^{\left(b\right)}=max\left\{l_{\infty }\left({\mathbb{C}}_{t}\right),t=1,\ldots ,T\right\}$ using the simulated data under pre-specified patterns.
4. Repeat the above steps B times, and we get the null distribution of W.

The raw p-value is calculated as

$$p=\frac{1}{B}\overset{B}{\underset{b=1}{\sum }}I\left(W^{\left(b\right)}>W\right).$$

We then apply the Holm-Bonferroni correction or BH procedure on raw p-values to control the FDR.

### ANCOM-BC2 for mixed effects models

Similar to the fixed effects model stated in (3), for each taxon j,j=1,…,d, and each sample i,i=1,…,n, suppose each sample has $n_{i}$ observations and $\sum_{i}n_{i}=n$. The offset-based mixed effects log-linear model is set up as

(26) $$y_{ij}=\theta_{i}1_{n_{i}}+X_{i}\beta_{j}+Z_{i}\alpha_{i}+\varepsilon_{ij},$$

where

1. $y_{ij}$ is the $n_{i}$-vector centered observed abundances,
2. $1_{n_{i}}=(1,\ldots ,1{)}^{T}\in {\mathbb{R}}^{n_{i}}$ is a vector of 1s,
3. $X_{i}$ is the $n_{i}\times p$ design matrix for fixed effects,
4. $\beta_{j}$ is the p-vector of fixed effects regression coefficients to be estimated,
5. $Z_{i}$ is the $n_{i}\times q$ design matrix for the random effects,
6. $\alpha_{i}$ is the q-vector random effects,
7. $\varepsilon_{ij}$ is the $n_{i}$-vector residuals.

The following distributional assumptions are made

$$\alpha_{i}∼N\left(0,D_{q\times q}\right),\;\varepsilon_{ij}∼N\left(0,\sigma_{j}^{2}I_{n_{i}}\right),\;\alpha_{i}⫫\varepsilon_{ij}\;\text{for}\;i=1,\ldots ,n.$$

Thus, for each taxon j,j=1,…,d, and each sample i,i=1,…,n, we have

$$y_{ij}∼N\left(\theta_{i}1_{n_{i}}+X_{i}\beta_{j},H_{ij}(\tau )\right),$$

where $H_{ij}(\tau )=Z_{i}DZ_{i}^{T}+\sigma_{j}^{2}I_{ni}$ (or $H_{ij}$ for short) denotes a general covariance matrix parametrized by τ.

Stack up observations across samples, we have:

(27) $$y_{j}=\theta +X\beta_{j}+Z\alpha +\varepsilon_{j},$$

where

$$\begin{array}{l} y_{j}=\left[\begin{array}{c} y_{1j}\\ y_{2j}\\ \vdots \\ y_{nj} \end{array}\right],\;\theta =\left[\begin{array}{c} \theta_{1}1_{n_{1}}\\ \theta_{2}1_{n_{2}}\\ \vdots \\ \theta_{n}1_{n_{n}} \end{array}\right],\;X=\left[\begin{array}{c} X_{1}\\ X_{2}\\ \vdots \\ X_{n} \end{array}\right],\;\beta_{j}=\left[\begin{array}{c} \beta_{j1}\\ \beta_{j2}\\ \vdots \\ \beta_{jp} \end{array}\right],\\ Z=\left[\begin{array}{cccc} Z_{1} & 0 & \ldots & 0\\ 0 & Z_{1} & 0 & 0\\ \vdots & \vdots & \ddots & \vdots \\ 0 & 0 & \ldots & Z_{1} \end{array}\right],\;\alpha =\left[\begin{array}{c} \varepsilon_{1j}\\ \alpha_{2}\\ \vdots \\ \varepsilon_{2j}\\ \vdots \\ \alpha_{n} \end{array}\right],\varepsilon_{j}=\left[\begin{array}{c} \varepsilon_{1j}\\ \varepsilon_{2j}\\ \vdots \\ \varepsilon_{nj} \end{array}\right]. \end{array}$$

That is,

$$y_{j}∼N\left(\theta +X\beta_{j},H_{j}(\tau )=\left[\begin{array}{cccc} H_{1j}(\tau ) & 0 & \ldots & 0\\ 0 & H_{2j}(\tau ) & 0 & 0\\ \vdots & \vdots & \ddots & \vdots \\ 0 & 0 & \ldots & H_{nj}(\tau ) \end{array}\right]\right),$$

where $H_{j}(\tau )$ (or $H_{j}$ for short) is a block diagonal matrix.

Similarly, we estimate θ and $\beta_{j}$ iteratively to obtain the corresponding preliminary estimators. As compared to Algorithm 1, the Maximum Likelihood (ML) is replaced with Restricted Maximum Likelihood (ReML)^52, 53^.

**Algorithm 3:** Iterative ReMLE

1:	**Initialize:**
	For *j=1,…,d*
	θ←0
	$y_{j}^{\left(\text{crt}\right)}\leftarrow y_{j}-\theta =y_{j}$
	$\beta_{j}\leftarrow \text{ReML}\left(y_{j}^{\left(\mathrm{crt}\right)}\right)=\text{ReML}\left(y_{j}\right)$
2:	**while** not converge **do**
3:	$\theta \leftarrow \frac{1}{d}\sum_{j=1}^{d}\left(y_{j}-X\beta_{j}\right)$
4:	$y_{\text{j}}^{\left(\text{crt}\right)}y_{\text{j}}-\theta$
5:	$\beta_{j}\leftarrow \text{ReML}\left(y_{\text{j}}^{\left(\text{crt}\right)}\right)$
6:	**end while**

Note that the estimators for regression coefficients $\beta_{j}$ and variance components τ are obtained iteratively by maximizing the following log-likelihood function:

(28) $$ℒ\left(\tau |y_{j}\right)=-\overset{n}{\underset{i=1}{\sum }}\text{log}\left|H_{ij}\right|-\overset{n}{\underset{i=1}{\sum }}\text{log}\left|{X_{i}}^{T}{H_{ij}}^{-1}X_{i}\right|-\overset{n}{\underset{i=1}{\sum }}{\left(y_{ij}-X_{i}\beta_{j}\right)}^{T}{H_{ij}}^{-1}\left(y_{ij}-X_{i}\beta_{j}\right),$$

where $\beta_{j}\leftarrow {\left(X^{T}{H_{j}}^{-1}X\right)}^{-1}X^{T}{H_{j}}^{-1}y_{j}$. As close-form solutions of (28) do not exist, Newton-Raphson method is usually employed^54^.

Suppose on convergence, $\theta \leftarrow \theta^{*},y_{j}^{\left(crt\right)}\leftarrow y_{j}^{{\left(crt\right)}^{*}},H\leftarrow H^{*},\beta_{j}\leftarrow \beta_{j}^{*}$, we have

$$\theta^{*}=\frac{1}{d}\overset{d}{\underset{j=1}{\sum }}\left(y_{j}-X{\beta_{j}}^{*}\right),\;y_{j}^{{\left(crt\right)}^{*}}=y_{j}-\theta^{*},\;{\beta_{j}}^{*}={\left(X^{T}H_{j}^{*-1}X\right)}^{-1}X^{T}H_{j}^{*-1}y_{j}^{{\left(crt\right)}^{*}}.$$

It is easy to show that there exists a vector $\delta \in {\mathbb{R}}^{p}$, such that

$$E\left(\theta^{*}\right)=\theta -X\delta ,\;E\left({\beta_{j}}^{*}\right)=\delta +\beta_{j}.$$

i.e., ${\beta_{j}}^{*}$ is a biased estimator for $\beta_{j}$.

Similar to the case of fixed effects model, we fit the Gaussian mixture model to each $\beta_{jk},k=1,\ldots ,p$ separately, to correct the bias δ, and final estimators for $\beta_{j}$ and θ are given by

$${\hat{\beta }}_{j}=\beta_{j}^{*}-{\hat{\delta }}^{EM},\;\hat{\theta }=\frac{1}{d}\overset{d}{\underset{j=1}{\sum }}\left(y_{j}-X{\hat{\beta }}_{j}\right).$$

Thus, for taxon j, the Wald statistic for the following hypotheses

$$H_{0}:A\beta_{j}=A{\beta_{j}}^{0},\;H_{1}:A\beta_{j}\neq A{\beta_{j}}^{0},$$

is given by

$$W_{j}={\left(A{\hat{\beta }}_{j}-A{\beta_{j}}^{0}\right)}^{T}{\left(A{\hat{\text{Σ}}}_{j}A^{T}\right)}^{-1}\left(A{\hat{\beta }}_{j}-A{\beta_{j}}^{0}\right)\;\;={\left(A{\beta_{j}}^{*}-A{\hat{\delta }}^{EM}-A{\beta_{j}}^{0}\right)}^{T}{\left(A{\hat{\text{Σ}}}_{j}A^{T}\right)}^{-1}\left(A{\beta_{j}}^{*}-A{\hat{\delta }}^{EM}-A{\beta_{j}}^{0}\right)\;\;\to_{d}\chi_{q}^{2},$$

where

1. ${\overset{ˆ}{\Sigma }}_{j}={\left(X^{T}{H_{j}}^{*-1}X\right)}^{-1}$,
2. q=rank(A).

To draw statistical inference under inequality constraints in linear mixed effects model, we use William’s type test statistic as specified in the last section, and adopt the Constrainted Linear Mixed Effects (CLME) framework^42^ into ANCOM-BC. The procedure is summarized as follows:

1. Obtain ${\overset{ˆ}{\beta }}_{j}$, the estimate of $\beta_{j}$ under the null hypothesis,
2. Compute the observed values of residuals and random effects: ${\overset{ˆ}{\varepsilon }}_{j}=(I-X{\left(X^{T}{\hat{H}}_{j}^{-1}X\right)}^{-1}X^{T}{\hat{H}}_{j}^{-1})y_{j}^{(crt{)}^{*}}$, and $\overset{ˆ}{\alpha }=\overset{ˆ}{\theta }Z^{T}{\hat{H}}_{j}^{-1}{\overset{ˆ}{\varepsilon }}_{j}$,
3. Standardize the observed values of the random effects and residuals. Define $\hat{a}=SE(\overset{ˆ}{\alpha }{)}^{-1}\overset{ˆ}{\alpha }$, and $\hat{e}=SE(\overset{ˆ}{\varepsilon }{)}^{-1}\overset{ˆ}{\varepsilon }$, where SE(⋅) denotes the standard error,
4. Obtain bootstrap samples. Let $a^{\left(b\right)}$ and $e^{\left(b\right)}$ denote the bootstrap samples of a and e, respectively. Then define ${\overset{ˆ}{\alpha }}^{\left(b\right)}=SE(\overset{ˆ}{\alpha })a^{\left(b\right)}$ and ${\overset{ˆ}{\varepsilon }}^{\left(b\right)}=SE(\overset{ˆ}{\varepsilon })e^{\left(b\right)}$. Finally construct the final bootstrap sample as: $y_{j}{\;}^{\left(b\right)}=\overset{ˆ}{\theta }+X{\overset{ˆ}{\beta }}_{j}+Z{\overset{ˆ}{\alpha }}^{\left(b\right)}+{\overset{ˆ}{\varepsilon }}^{\left(b\right)}$,
5. Repeat B times (b=1,…,B), we construct the null distribution for the test statistic.
6. Compute raw p-values,
7. Apply the Holm-Bonferroni correction (default) or BH procedure on raw p-values to control the FDR.

## Figures and Tables

**Table 1.** Summary of notations

Notation	Description

i	Sample index, i=1,2,…,n.
j	Tax on index, j=1,2,…,d.
k	Index of fixed effects, k=1,2,…,p.
l	Index of Random effects, l=1,2,…,q.
$x_{ik}$	The $k^{th}$ fixed effect of interest for the $i^{th}$ sample.
$z_{il}$	The $l^{th}$ random effect of interest for the $i^{th}$ sample.
$A_{ij}$ ‡	Unobserved abundance of $j^{th}$ taxon in a unit volume of ecosystem of $i^{th}$ sample.
$O_{ij}$ ‡	Observed abundance of $j^{th}$ taxon in a random specimen taken from a unit volume of ecosystem of $i^{th}$ sample.
$E_{ij}$ ‡	Random error for taxon j in sample i.
$S_{i}$ †	Sample-specific sampling fraction.
$C_{j}$ †	Taxon-specific sequencing efficiency.
$a_{ij}$ ‡	log $A_{ij}$.
$o_{ij}$ ‡	log $O_{ij}$.
$e_{ij}$ ‡	Random error for taxon j in sample i in log scale.
$s_{i}$ †	Sample-specific sampling fraction in log scale.
$c_{j}$ †	Taxon-specific sequencing efficiency in log scale.

† Parameter;

‡ Random variable.

## Data Availability

Simulation data can be found on the corresponding GitHub repository^55^. The soil microbiome data for aridity can be found in Qiita: https://qiita.ucsd.edu/study/description/10360. The gut microbiome data for IBD can be found in Qiita: https://qiita.ucsd.edu/study/description/11546.
